# Effectiveness of a guided e-health sleep and biological clock intervention in university students (i-Sleep & BioClock) – results of a randomized controlled trial

**DOI:** 10.1192/j.eurpsy.2025.1442

**Published:** 2025-08-26

**Authors:** L. M. Pape, A. van Straten, S. Struijs, P. Spinhoven, N. Antypa

**Affiliations:** 1 Department of Clinical Psychology, Leiden University, Leiden; 2 Department of Clinical, Neuro- & Developmental Psychology, Vrije Universiteit Amsterdam, Amsterdam, Netherlands

## Abstract

**Introduction:**

Sleep problems are very prevalent among university students, affecting their mood, energy levels, daily functioning, and quality of life. Irregular sleep-wake patterns contribute to the disruption of their circadian rhythms. Cognitive behavioural therapy for insomnia (CBT-I) has been proven effective in adults, but research in university students is still limited.

**Objectives:**

The aim of this study is to investigate the effectiveness of a guided digital self-help intervention targeting sleep and the biological clock in university students to improve sleep and mental health outcomes.

**Methods:**

We conducted a two-arm randomized controlled trial in nine Dutch Universities. We included 196 university students (Bachelor, Master, and PhD) with self-reported insomnia symptoms (Insomnia Severity Index ≥ 10) and randomly assigned them (1:1) to receive the 5-week ‘i-Sleep & BioClock’ intervention or online psychoeducation. The intervention is based on CBT-I with specific emphasis on the biological clock. It consists of 5 weekly online modules and is guided by online coaches. The primary outcome is insomnia severity. Secondary outcomes are depression, anxiety, daily functioning, academic performance, quality of life, and sleep & light exposure diary outcomes. Outcomes were measured at baseline, mid-treatment (3 weeks), post-treatment (6 weeks after baseline), and at 18 weeks follow-up. Data will be analyzed with intention-to-treat analysis using linear mixed models. The study is registered at ClinicalTrials.gov (NCT06023693).

**Results:**

Recruitment started on Nov 1^st^, 2023 and ended on Sept 5^th^, 2024. Data collection is currently ongoing and will be finalized in January 2025. We hypothesize that the guided digital self-help intervention will reduce insomnia severity and improve mental health outcomes in students (results will be presented).

**Conclusions:**

Findings from this randomized controlled trial will contribute to the growing body of knowledge on digital sleep interventions and their potential impact on mental health. If the intervention proves effective, we aim to disseminate the intervention widely in higher education to benefit a broader student population.

**Disclosure of Interest:**

None Declared

